# Fingolimod Potentiates the Antifungal Activity of Amphotericin B

**DOI:** 10.3389/fcimb.2021.627917

**Published:** 2021-04-23

**Authors:** Lu-Qi Wei, Jing-Cong Tan, Yue Wang, Yi-Kun Mei, Jia-Yu Xue, Lei Tian, Ke-Yu Song, Lu Han, Ying-Chao Cui, Yi-Bing Peng, Jing-Quan Li, Ning-Ning Liu, Hui Wang

**Affiliations:** ^1^ State Key Laboratory of Oncogenes and Related Genes, Center for Single-Cell Omics, School of Public Health, Shanghai Jiao Tong University School of Medicine, Shanghai, China; ^2^ Department of Laboratory Medicine, Ruijin Hospital, Shanghai Jiao Tong University School of Medicine, Shanghai, China; ^3^ Faculty of Medical Laboratory Science, Shanghai Jiao Tong University School of Medicine, Shanghai, China

**Keywords:** *Candida albicans (C. albicans)*, antifungal agents, amphotericin B (AMB), fingolimod (FTY720), reactive oxygen species (ROS)

## Abstract

*Candida albicans* (*C. albicans*) is an opportunistic human fungal pathogen that can cause severe infection in clinic. Its incidence and mortality rate has been increasing rapidly. Amphotericin B (AMB), the clinical golden standard antifungal agent, has severe side effects that limit its clinical application. Thus, lowering the concentration and increasing the efficacy of AMB in a combinatorial antifungal therapy have been pursued by both industry and academia. Here we identify that fingolimod (FTY720), an immunomodulatory drug used for oral treatment of relapsing-remitting multiple sclerosis, can potentiate the efficacy of AMB against *C. albicans* growth synergistically. Furthermore, we observe an antifungal efficacy of FTY720 in combination with AMB against diverse fungal pathogens. Intriguingly, cells treated with both drugs are hypersensitive to endothelial endocytosis and macrophage killing. This is later found to be due to the hyperaccumulation of reactive oxygen species and the corresponding increase in activities of superoxide dismutase and catalase in the cells that received combinatorial treatment. Therefore, the combination of AMB and FTY720 provides a promising antifungal strategy.

## Introduction

As one of the most important human fungal pathogens, *C. albicans* normally resides in human body including oral cavity, gastrointestinal (GI) tracts and vagina as a non-pathogenic commensal organism (Gulati and Nobile, 2016; Prieto et al., 2016). However, it will cause severe mucosal and even systemic candidiasis infections when the microbial homeostasis is disrupted (Achkar and Fries, 2010; Gulati and Nobile, 2016; Prieto et al., 2016). The mucosal injury in GI tracts caused by diseases such as IBD progression facilitates the colonization and invasion of *C. albicans* leading to higher frequency of GI tracts infection (Gerard et al., 2015; Li et al., 2018; Stamatiades et al., 2018). However, the arsenal of antifungals is rather limited compared with that of antibiotics. The polyene macrolide antifungal amphotericin B (AMB) is very effective in the treatment of systemic candidiasis, and is recommended for IBD patients with opportunistic fungal infections (Belenky et al., 2013). However, due to its poor permeability across the membrane, an overdose of AMB must be administered to the patients in clinic, resulting in severe side effects such as nephrotoxicity (Mesa-Arango et al., 2014). To minimize the side effects, AMB was combined with other antifungals such as azoles (Johnson et al., 2004; Mukherjee et al., 2005). However, frequent application of azoles and their derivatives may lead to increased drug resistance (Sarkar et al., 2014). Thus, it is necessary to investigate the combinatorial application of AMB and other compounds to reduce the dosage of AMB while maintaining or increasing its efficacy.

Fingolimod (FTY720) is a S1P receptor inhibitor against several pro-inflammatory conditions including multiple sclerosis (Sukocheva et al., 2020). It can reduce the severity of UC, attenuate intestinal injury, and shows anticancer effects in GI tract (Feng et al., 2018; Sukocheva et al., 2020). When combined with AMB, FTY720 was thus identified to be a promising candidate which exerts a synergistic effect with AMB against the growth of *C. albicans*. FTY720, an agonist of sphingosine 1-phosphate receptor (SIPR) and a substrate for sphingosine kinase (SphK), is an immunomodulatory drug used for oral treatment of relapsing-remitting multiple sclerosis (RRMS) (Huwiler and Zangemeister-Wittke, 2018), which has little interference with immune response to infection (Pinschewer et al., 2000). In this study, we evaluated the antifungal activity of FTY720 alone and in combination with AMB against *C. albicans*. We further tested the inhibitory efficacy of the drug combination against the growth of diverse fungal species and unveiled the mechanisms underlying the synergistic effect.

## Materials and Methods

### Strains, Medium and Growth Condition

Details of strains used in this study are shown in **Table S1**. The fungal cells were cultured in YPD medium (1% yeast extract, 2% glucose and 2% peptone) and grown for 24 hours at 30°C. FaDu cells were cultured in MEM (Thermo Fisher) supplemented with 1% Penicillin-Streptomycin (Gibco, Cat# 15070063) and 10% fetal bovine serum (FBS) (BI, Cat# 1545515), and RAW264.7 cells were cultured in DMEM supplemented with 10% FBS (BI, Cat# 1545515). FTY720 (Sigma, Cat# SML0700) and AMB (Sigma, Cat# V900919) was diluted in fresh YPD medium or cell culture medium to get the indicated concentration and the control group contained equivalent concentration of DMSO.

### Growth Curve Assay

Cells grown overnight in YPD at 30°C were diluted to OD_600_ = 0.2 (0.1 OD_600_ for clinically isolated Candida strains) with different drug treatment in flat-bottomed 96-well plate. The OD_600_ was obtained every 60 min in a BioTek Synergy H1 Multi-Mode Microplate Reader (Winooski, VT, USA). The SDs of at least three technical replicates were calculated and graphed in Graphpad Prism Software. All panels shown represent at least three biological replicates.

### Determination of Minimum Inhibitory Concentrations

The minimum inhibitory concentrations (MIC) of FTY720 alone and the combination with AMB against *C. albicans* isolates were determined according to the broth microdilution method described in the CLSI guidelines (Clinical Laboratory Standards Institute, 2008). Growth inhibition was determined by visual reading and optical densities measured at 600 nm using a BioTek plate reader. The MIC_80_ was defined as 80% growth inhibition compared to a no-drug control (Lewis et al., 2002; Li et al., 2011; Khan and Ahmad, 2012). The *in vitro* interaction between two drugs was defined by the fractional inhibitory concentration index (FICI) (Odds, 2003). The FICI model was expressed as follows: FICI = FIC_AMB_ + FIC_FTY720_ = (MIC_80_ of AMB in combination/MIC_80_ of AMB alone) + (MIC_80_ of FTY720 in combination/MIC_80_ of FTY720 alone). The interpretation of FICI was: FICI for synergy ≤ 0.5, FICI for antagonism > 4.0, and 0.5 <FICI ≤ 4.0 without interaction.

### Effect of AMB and FTY720 Combination on *C. albicans* Biofilm Formation

The cells were diluted into 100 µL RPMI-1640 medium in a 96-well plate and incubated in a 37°C incubator for 4 hours to form an adherent biofilm. The biofilm was treated with FTY720 and AMB for 24 hours. To quantify biofilm formation, XTT (Sangon Biotech, Cat# A602525-0250) solution containing 1% phenazine methyl sulfate (Sigma-Aldrich, USA) was added to each well (100 µl) and incubated at 37°C for 2 – 3 h. The absorbance of the supernatant was then measured at 492 nm using a BioTek microplate reader (Li et al., 2019).

### Filamentation Assay

Cells grown overnight were diluted with hyphae inducing medium (YPD+10% serum, M199 and Spider medium) containing either FTY720 or AMB to an OD_600_ of 0.05. After incubation at 37°C for 2 h, the cells were observed under the NIKON microscope and compared with the vehicle control. All experiments were performed in triplicates.

### Colony Forming Units (CFUs) Assay

*C. albicans* cells grown overnight in YPD for 15 hours were diluted to OD_600_ = 0.2 in YPD and OD_600_ = 0.05 in YPD + 10% serum medium. At 0 h, the cells were diluted and plated onto YPD plates. After treatment with 11 mg/L FTY720 in YPD at 30°C or in YPD+10% serum at 37°C for 2 h, the cells were diluted and plated onto YPD plates. The CFUs were counted after 24 h of incubation at 30°C. The percent survival was calculated as (CFUs after FTY720 treatment/CFUs at 0 h) × 100.

### ROS Measurement in *C. albicans*

*C. albicans* cells grown overnight in YPD for 15 hours were adjusted to 1×10^7^ cells/ml. The intracellular level of ROS was measured with the fluorescent dye 2′,7′-dichlorodihydrofluorescein diacetate (H_2_DCFDA) (Sigma, Cat# D6883). The dye was added into the medium to a final concentration of 50 μM. After incubation with the dye for 45 min at 37°C, the cells were washed with PBS and exposed to the drugs and incubated at 30°C with constant shaking (200 rpm) for 2 hours. Cell samples were observed with confocal scanning laser microscope with excitation wavelength at 485 nm and emission wavelength at 520 nm. At the same time, cell suspensions were harvested and transferred to the wells of a flat-bottom microplate (BMG Microplate, 96 well, Black) to detect fluorescence intensity by a BioTek microplate reader with excitation wavelength at 485 nm and emission wavelength at 520 nm and the ratio of intensity normalized with OD_600_ of the culture was calculated (Liu et al., 2018). For the ROS clearance assay by N-acetyl-l-cysteine (NAC) (Sigma, Cat# A9165) (Carter et al., 2005; Kim et al., 2013), cells were grown overnight in YPD at 30°C and diluted to OD_600_ = 0.1 with different drug treatments in flat-bottomed 96-well plate. The statistical significance of OD_600_ at 8 hours (in the cell logarithmic phase) was calculated using ordinary one-way ANOVA with Dunnett’s corrected post-hoc comparisons. All panels shown represent at least three biological replicates. At least three biological replicates were obtained for each experiment.

### SOD Activity and Catalase Activity Measurement

*C. albicans* cells grown to exponential phase in YPD medium overnight were collected by centrifugation and washed twice with PBS with final OD_600_ = 0.1 in YPD medium. The cells were then exposed to DMSO or 5.5 mg/L FTY720 or 0.08 mg/L AMB or the drug combination at 30°C with constant shaking (200 rpm) for 3 hours. The following steps were completed on ice: after incubation, cells were harvested, washed twice with PBS, resuspended with prepared lysis buffer and transferred to tubes containing silicon beads (0.1 mm Zirconia/Silica Beads, Biospec, catalog # 11079101z). After beads-beating at 3000 rpm for 45 s per cycle for 4 cycles (Chowdhury and Kohler, 2015; Liu et al., 2017), the supernatants were determined for enzymatic activity after centrifugation at 12000 rpm for 10 minutes at 4°C. Protein concentration was measured using the Pierce™ BCA Protein Assay Kit (Thermo Scientific #23250). SOD activity of the cell lysate was then detected with the SOD Assay Kit - WST (DOJINDO #S311) and the absorbance of the samples was measured with a BioTek microplate reader at 450 nm at 37°C to calculate its SOD activity. Catalase activity was measured by a colorimetric catalase assay (Beyotime #S0051) according to the manufacturer’s instructions (Salvatori et al., 2018).

### Lactate Dehydrogenase (LDH) Assay

To evaluate the cytotoxicity of FTY720, a total number of 2 × 10^4^ FaDu cells (or 1 × 10^4^ NCM460 cells) were incubated with different concentration of FTY720 for 1 h in MEM (or DMEM) medium containing 10% FBS (BI Cat#1545515) in 37°C incubator with 5% CO_2_. The LDH level was evaluated using Cytotoxicity LDH Assay Kit (Dojindo Cat#CK12) according to manufacturer’s instructions (Mei et al., 2020).

### Measurement of Host Cell Endocytosis

To determine the effects of drug combination on endocytosis, the FaDu cells were incubated with 1 × 10^5^ yeast-phase *C. albicans* cells per well for 45 min in MEM medium containing 1% Penicillin-Streptomycin (Gibco, Cat#15070063) and 10% FBS (BI, Cat#1545515) under different drug treatments in a 37°C incubator with 5% CO_2_. The cells were then diluted and plated onto YPD plate for colony forming units (CFUs) count.

### Macrophage Killing Assay

Macrophages were co-incubated with *C. albicans* (MOI = 1) at 37°C for 2 h in an incubator containing 5% CO_2_. Cells were washed adequately in cold PBS, resuspended in warm DMEM medium, and further incubated at 37°C with drug supplementation for 2 hours. Macrophages cells were lysed in PBS containing 0.1% Triton X-100 to release fungal cells. A serial dilution was performed and plated onto YPD plates. The CFUs were counted after 24 h of incubation at 30°C. The percent survival was calculated as (1- CFUs after co-culture with macrophages/CFUs of *C. albicans* cultured with medium without macrophages) ×100 (Vonk et al., 2002; Liu et al., 2018; Salvatori et al., 2018).

### Statistics

Statistical analysis for comparisons of numerical values was made using SPSS software (Version 20., Chicago, IL, USA). The calculations were performed by the GraphPad Prism statistical program (GraphPad Software, Inc., CA, USA).

## Results

### Inhibitory Effect of the Combination of FTY720 and AMB Against Multiple Fungal Species

**Figure 1:**
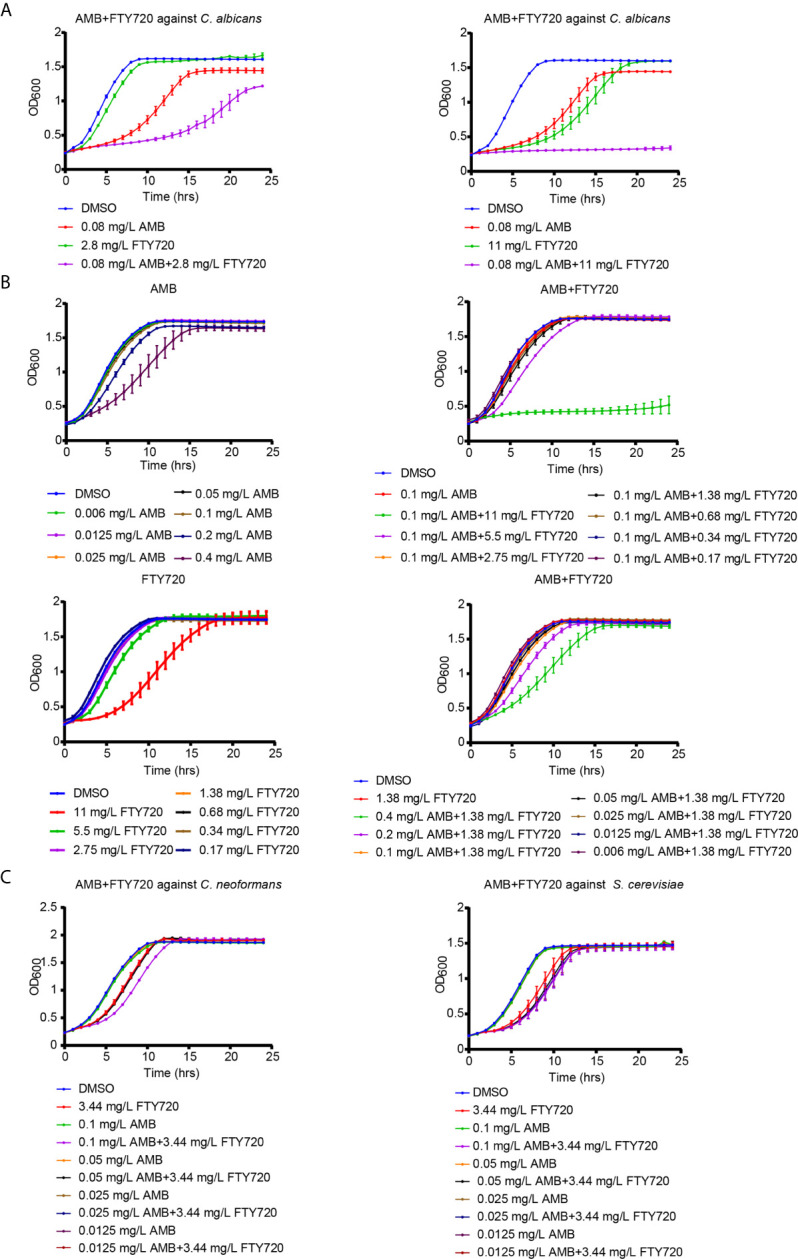
Inhibitory effect of FTY720 and AMB on multiple fungal species. **(A, B)** Growth curve analysis of *C. albicans* in the presence of AMB and FTY720 at different concentration. The OD_600_ was obtained every 60 min in a BioTek plate reader. **(C)** Dose-dependent growth inhibition of *S. cerevisiae* and *C. neoformans* by FTY720 and AMB. Results are represented by the mean ± SD of three biological replicates.

FTY720 exhibited a promising combinatorial effect to inhibit the growth of *C. albicans* when combined with AMB (**Figure 1A**). To further confirm whether it is synergistic or not, we performed the broth microdilution method to calculate the MIC_80_ and FICI of AMB and FTY720. MIC_80_ represents the minimum inhibitory concentration of drug that inhibited fungal growth by 80% compared with control. The combination of FTY720 and AMB exhibits an enhanced efficacy to suppress *C. albicans* growth in comparison with single FTY720 or AMB treatment. The FICI was calculated to be 0.19 < 0.5 which indicated a synergistic effect (**Table 1** and **Figure 1**). Thus, we demonstrated that the combination of FTY720 and AMB indeed exerts a synergistic inhibitory effect against *C. albicans* (**Figure 1B**).

**Table 1:** Drugs interactions of FTY720 and AMB against *C. albicans in vitro*.

Strains	MIC_80_ (mg/L)^a^				FICI^b^	Activity
	Alone		Combined			
	FTY720	AMB	FTY720	AMB		
SC5314	> 22	0.32	1.38	0.04	0.19	SYN

a FICI, fractional inhibitory concentration index

b SYN, synergism;

MIC_80_ values and FICIs were calculated from three independent experiments.

To test the antifungal activity against multiple fungal pathogens, we further treated *Cryptococcus neoformans* (*C. neoformans*) and *Saccharomyces cerevisiae* (*S. cerevisiae*) with the combination of FTY720 and AMB. It was shown that there also exists a more significant inhibitory effect for the combination against *C. neoformans* and *S. cerevisiae* comparing with single treatment (**Figure 1C** and **Figure S1**), which indicated that the drug combination presents antifungal activity against multiple fungal pathogens.

### The Drug Combination Inhibits the Growth of Clinically Isolated Candida Strains

**Figure 2:**
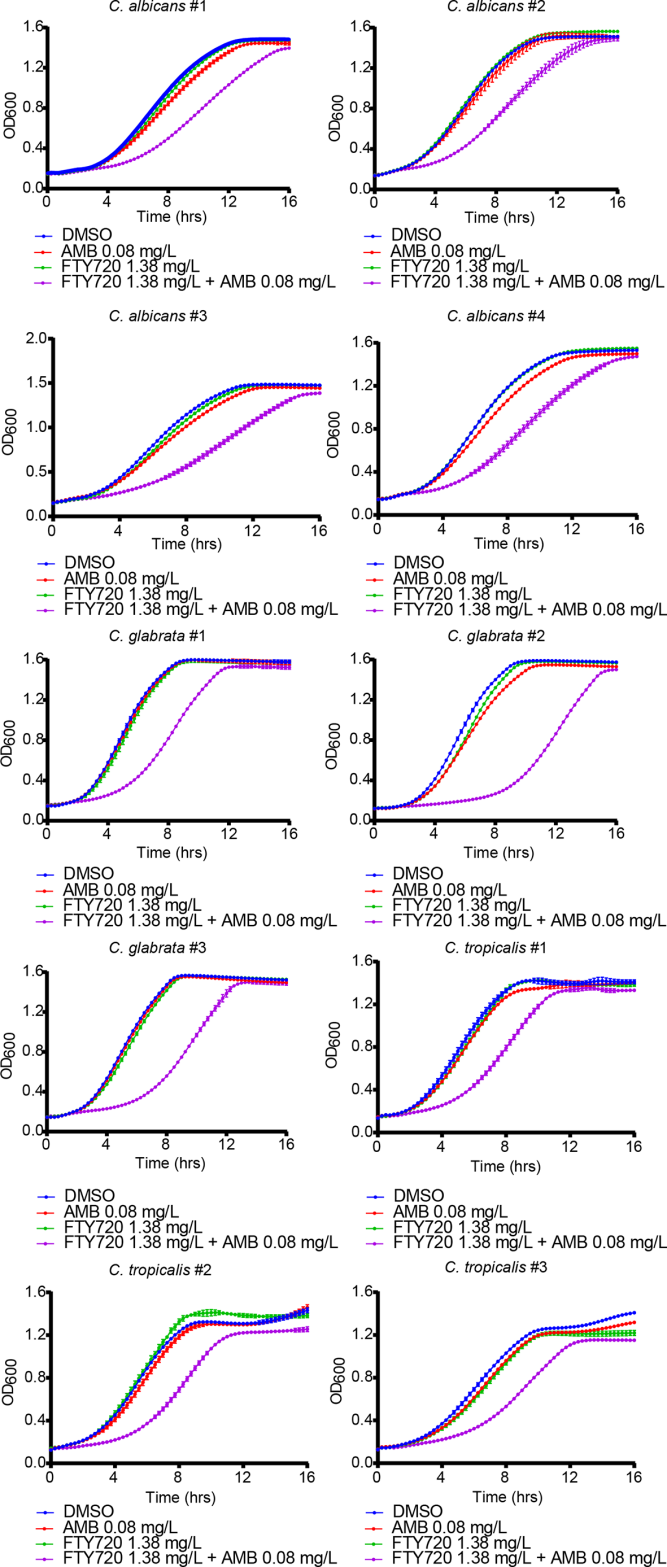
Enhanced inhibitory effect of FTY720 and AMB on different *Candida* clinical isolates. The OD_600_ was obtained every 15 min in a BioTek plate reader for 16 hours. Results are represented by the mean ± SD of three biological replicates.

To further confirm the inhibitory effect of combination on *Candida* cells, we isolated four strains of *C. albicans*, three strains of *Candida glabrata* (*C. glabrata*) and three strains of *Candida tropicalis* (*C. tropicalis*) from the respiratory tract, urinary tract, and blood samples of the patients in the clinical laboratory of Ruijin Hospital, Shanghai, China. As shown in **Figure 2** and **Figure S2**, the growth of clinically isolated *C. albicans*, *C. glabrata*, and *C. tropicalis* under combinatorial treatment was significantly inhibited. Thus, FTY720 also potentiates the antifungal effect of AMB against clinical *Candida* isolates.

### Suppression of Hyphal Growth and Biofilm Information by the Drug Combination in *C. albicans*

**Figure 3:**
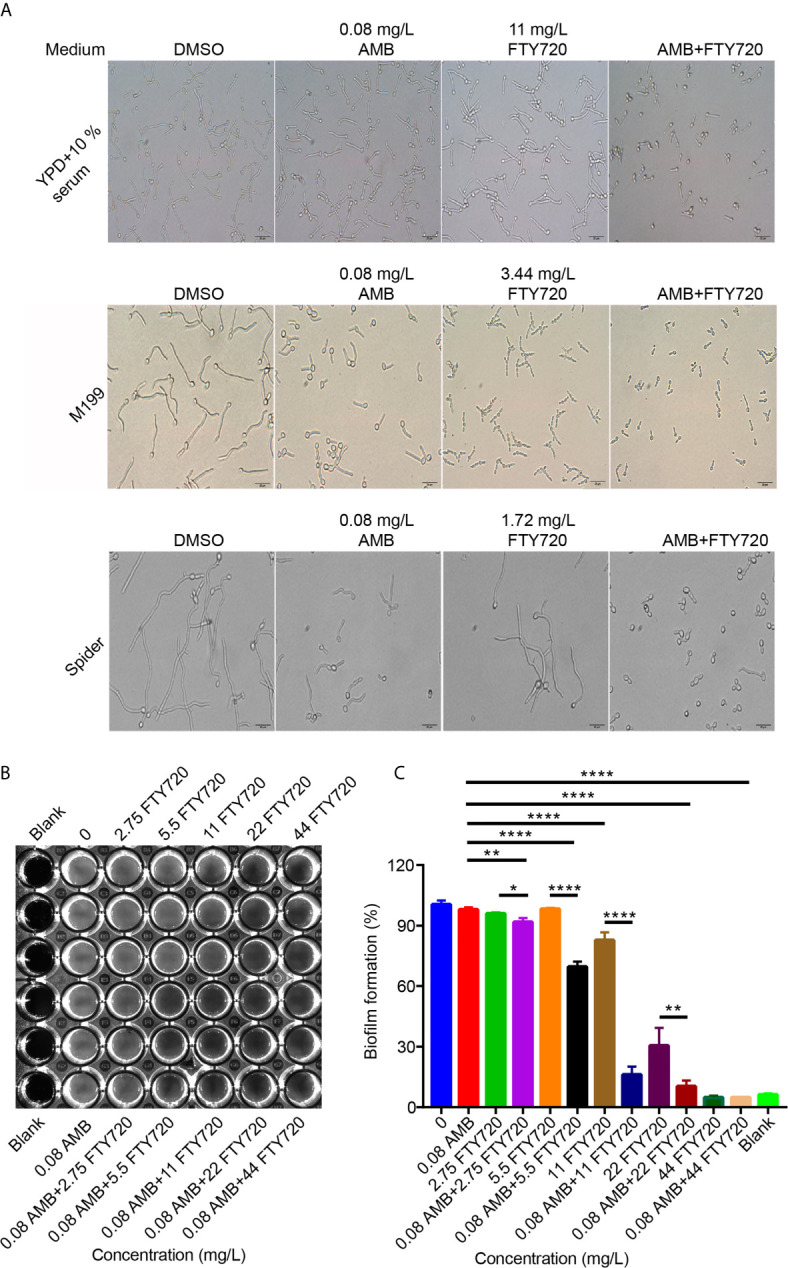
Suppression of *C. albicans* filamentation and biofilm formation by FTY720 and AMB. **(A)** Cells were inoculated into different hyphae-inducing medium in the presence or absence of FTY720 and AMB and grown for 2 h at 37°C (Scale bar=20 μm). **(B)** Adhesive biofilm formation of *C. albicans* in RPMI medium supplemented with different concentrations of FTY720 and AMB in a 96-well plate and photos were taken after discarding supernatant. **(C)** *In vitro* activity of different concentrations of FTY720 and AMB at the early stage of biofilm formation after incubation in 96-well plate at 37°C for 4 h, as determined by XTT colorimetric readings at 490 nm. Error bars represent SDs of 3 technical replicates. Statistical significance was determined using one-way analysis of variance (ANOVA) with Tukey’s corrected post-hoc comparisons. *P < 0.05, **P < 0.01, ****P < 0.0001.

The ability to switch between hyphae and yeast is critical for *C. albicans* pathogenesis. Next, we asked whether the hyphal growth would be suppressed by FTY720 combined with AMB. In YPD medium supplemented with 10% serum, single treatment by FTY720 didn’t change the morphology of *C. albicans* (**Figure 3A**). However, the filamentation was inhibited upon addition with the drug combination. In both M199 and Spider medium, the hyphal length was much shorter than that of vehicle control when treated with FTY720 alone. Surprisingly, the filamentation was substantially suppressed by combinatorial treatment with both FTY720 and AMB (**Figure 3A**). These results indicate that combination of FTY720 and AMB exhibits a much stronger inhibitory effect on the filamentation of *C. albicans* than the single treatment with FTY720 or AMB.

To confirm the possibility that the cells are not able to filament because their growth is inhibited, we performed the CFUs assay to determine whether the cell growth is inhibited or not. We found that they were decreased in both YPD and YPD + 10% serum media after treatment with FTY720 for 2 hours (**Figure S3**). Thus, our results indicated that the cells were not able to filament might be due to growth inhibition.

Additionally, AMB and FTY720 significantly inhibited the biofilm formation in *C. albicans* (**Figures 3B, C**). To further assess the inhibitory activity of the drug combination on *C. albicans* biofilm, we used an XTT colorimetric assay that can monitor the metabolic activity of the biofilm (Jin et al., 2003; Li et al., 2019). Interestingly, combined treatment of AMB and FTY720 resulted in a significant reduction in biofilm adhesion (**Figure 3C**). Taken together, these results demonstrated that combination of FTY720 and AMB inhibits biofilm formation significantly.

### ROS Hyperaccumulation After Treatment With Both FTY720 and AMB in *C. albicans*

**Figure 4:**
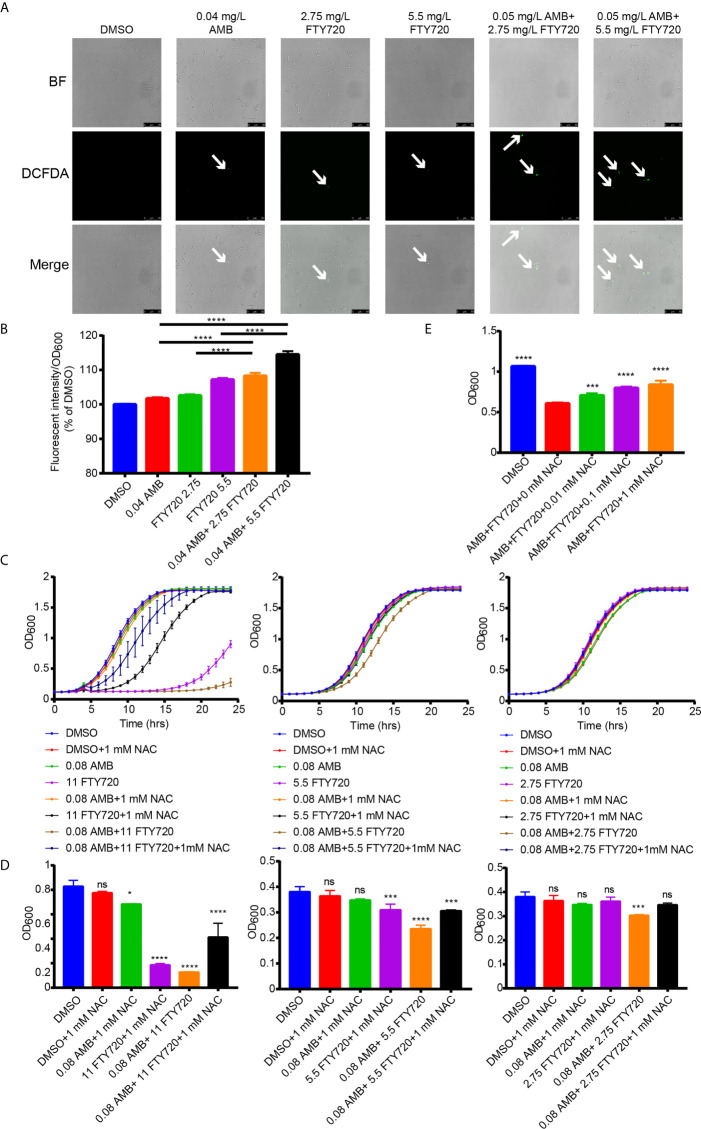
Combination of FTY720 and AMB induced ROS hyperaccumulation and increased activity of SOD and CAT in *C. albicans*. **(A)** ROS generation in *C. albicans* cells stimulated by 0.04 mg/L of AMB and 5.5 or 2.75 mg/L of FTY720 loaded with 50 µM fluorescent dye, 2′,7′-dichlorodihydrofluorescein diacetate (H_2_DCFDA) (BF, brightfield). **(B)** Fluorescent intensity of ROS in *C. albicans* cells treated by 0.04 mg/L AMB and 5.5 or 2.75 mg/L FTY720. **(C)** Cells were treated with single or combination of 0.08 mg/L AMB and 11, 5.5, or 2.75 mg/L FTY720 with/without 1mM NAC. The OD_600_ was obtained every 60 min in a BioTek plate reader for 24 hours. **(D)** Cell growth was quantified after incubation for 8 hours. **(E)** Cell growth was quantified after treatment with single or combination of 0.08 mg/L AMB and 2.75 mg/L FTY720 with/without various concentrations of NAC. Results are represented by the mean ± SD of three biological replicates. *P < 0.05 *vs* control, ***P < 0.001 *vs* control, ****P < 0.0001 vs control. ns, not significant vs control.

ROS production is one of the important mechanisms contributing to the fungicidal effect of AMB (Mesa-Arango et al., 2014). To detect the ROS levels, the cells were incubated with 2’, 7’-dichlorodihydrofluorescein diacetate (H_2_DCFDA). As shown in **Figure 4A**, we observed an increased fluorescence induced by single treatment with AMB or FTY720. However, when combined with FTY720, the ROS levels in *C. albicans* was significantly elevated upon treatment with the combination of AMB and FTY720 (**Figure 4B**). Furthermore, while 0.04 mg/L AMB didn’t result in significant increase in fluorescence, the increasing concentration of FTY720 significantly increases florescence in a dose-dependent manner, compared with single treatment. Oxidative stress induced by accumulation of ROS was reported to stimulate the antioxidant enzyme activity including SOD and CAT in cells (Guirao-Abad et al., 2017). As shown in our results, the relative antioxidant enzymatic activities of SOD and CAT in *C. albicans* after treatment with AMB/FTY720 was significantly increased more than that of the single agent treatment (**Figures S4A, B**).

AMB has been shown to play a fungicidal role by inducing ROS generation (Guirao-Abad et al., 2017). We hypothesized that clearance of ROS by NAC would thus eliminate the hypersensitivity to the combinatorial treatment. Thus, we further investigated the scavenge of both intracellular and extracellular ROS by N-acetyl-l-cysteine (NAC) and tested if the elimination of ROS will weaken the inhibitory activity of AMB and FTY720. As shown in **Figures 4C–E**, the addition of 1 mM NAC dramatically compromised the inhibitory effects of multiple AMB and FTY720 combination. Higher concentrations of NAC were more significant on attenuating the fungicidal effects of drug combination (**Figure S5**). Collectively, we demonstrated that combination of FTY720 and AMB induced ROS hyperaccumulation in *C. albicans* leading to cell growth defect.

### Low Concentration of FTY720 Showed No Cytotoxicity on FaDu and NCM460

We evaluated the cytotoxicity of FTY720 on two mammalian cell lines *via* LDH assay, FaDu (hypopharyngeal carcinoma cell line) and NCM460 (normal human colon mucosal epithelial cell line). As shown in **Figure S6**, we observed a dose-dependent cellular damage by FTY720 on both cell lines. While the higher concentration (88 mg/L, 44 mg/L and 22 mg/L) of FTY720 induced significant cellular damage, lower dosage (11 mg/L and below) showed no cytotoxicity, suggesting the potential application of low concentration of FTY720 as non-toxic antifungal agent (**Figure S6**).

### The Endocytosed *C. albicans* Was Reduced After Treatment of FTY720 and AMB

The hyphal invasion by *C. albicans in vitro* can severely damage endothelial cells or oral epithelial cells. Endocytosis of the pathogen is prone to induce host cell damage (Martinez-Lopez et al., 2006). Therefore, we determined the number of *C. albicans* cells adhering to the oral epithelial cell line FaDu and investigated the endocytosis of *C. albicans* by these host cells after treatment with FTY720 and AMB (**Figure S7A**). FTY720/AMB combination showed almost 58% reduction in the number of colonies visualized by CFU counts that were endocytosed by oral epithelial cells compared to the no drug control. Furthermore, cells treated with FTY720 or AMB alone was also hypersensitive to epithelial endocytosis, which suggested that FTY720 and AMB disrupted the endocytosis by epithelial cells. In aggregate, these results indicate that *C. albicans* treated with FTY720 and AMB cannot invade oral epithelial cells efficiently, and the drug combination treatment can reduce the ability of *C. albicans* to destroy cells (*P* <0.001).

### Increased Macrophage Killing of *C. albicans* Cells Treated With FTY720 and AMB

As macrophages are the major innate immune defense against *C. albicans* invasion, we then measured macrophage killing of fungal cells by RAW264.7. Less *C. albicans* released by the lysed macrophages were observed with AMB alone (*P* <0.01) than with FTY720 alone (*P* <0.05) (**Figure S7B**). The combination of FTY720 and AMB exhibited the most significant antifungal efficacy against *C. albicans* because the *C. albicans* cells treated by FTY720 combined with AMB were more sensitive to the phagocytic cell killing than vehicle control (*P* < 0.001), which is consistent with the conclusions drawn from our previous experiments. Collectively, these results concluded that the combination of FTY720 and AMB exerted a stronger inhibitory effect on *C. albicans* than the single drug treatment.

## Discussion

AMB is the gold standard antifungal drug for most of the severe invasive fungal infections, especially in *Candida* spp. infections of IBD patients. However, the serious adverse effects like nephrotoxicity limited its clinical application especially during the early phase of treatment (Mesa-Arango et al., 2014). Development of new antifungal agents will be costly and time-consuming. Drug repurposing provides an immediate therapeutic strategy against fungal infection (Mei et al., 2020). Furthermore, the combinatory strategy which enhances the efficacy and reduces the dosage of AMB to lessen the severity of the side effects are emerging in the industry. And the combination of commercial antifungal agents with an FDA approved drug with antifungal activity may be much easier to approach to the clinical practice (Nooney et al., 2005). In this study, we found that FTY720 remarkably inhibited the growth of *C. albicans* in combination with AMB compared with AMB alone. The FICI index indicated a synergistic interaction between AMB and FTY720 (**Table 1**), which is reported for the first time as far as we know.

FTY720 was reported to improve the symptoms of ulcerative colitis (UC) (Feng et al., 2018; Sukocheva et al., 2020). However, the mechanism of its beneficial effects in colitis remains unknown (Danese et al., 2018; Panés and Salas, 2018). Our study suggested its potential on eliminating the overgrowth of *C. albicans*.

The filamentation was almost completely inhibited by the combination of FTY720 and AMB (**Figure 3A**). Pathogenic biofilms composed of disrupted microbiota are capable of exacerbating intestinal inflammation in IBD progression (Hager and Ghannoum, 2017). *Candida* biofilms, composed of a hydrophilic matrix cells and hyphae, have unique hydrophobic structure. AMB binds directly to ergosterol to alter cell membrane activity in fungal cells (Ghannoum and Rice, 1999). Since filamentation is an important feature of biofilm formation (Nobile and Johnson, 2015), we hypothesized that the drug combination may have an inhibitory effect on biofilm formation. Indeed, we intuitively monitored the disappearance of biofilms in the presence of different concentrations of drug combinations (**Figures 3B, C**). Thus, this drug combination is potential to improve the treatment of *Candida* biofilm associated infection.

Combinatorial treatment with AMB and FTY720 results in a remarkably increased ROS level in *C. albicans*. ROS normally includes oxygen anions, free radicals and peroxide (Scherz-Shouval and Elazar, 2007). Remarkable accumulation of ROS can result in oxidative stress and cellular components damage which are toxic to the organisms, even leading to autophagy, apoptosis or necrosis (Scherz-Shouval and Elazar, 2007; Nakagawa, 2008; Yu et al., 2016). AMB binds to ergosterol at plasma membrane causing loss of ions, induces ROS accumulation in different pathogenic fungal species (Mesa-Arango et al., 2014; Silva et al., 2020) and causes lipid peroxidation through a greatly increased ROS level and reactive nitrogen species (NOS) level (Ferreira et al., 2013). The ROS induction subsequently leading to apoptosis is an important mechanism of AMB in *C. albicans* (François et al., 2006). After eliminating ROS in the environment by NAC, the growth under drug treatment become similar to the control group. It was shown that the fungicidal effects of drugs were impaired by reducing the drug-induced ROS amount, indicating that ROS accumulation may be one of the mechanisms underlying the antifungal drug combination.

ROS generation stimulated antioxidant pools including non-enzymatic and enzymatic antioxidizing agents, such as SOD, CAT and peroxidases, to counteract its detrimental effects and reduce the level of ROS (Scherz-Shouval and Elazar, 2007; Yu et al., 2016), which plays a key role in the antioxidant defense system for aerobic organisms (Dantas Ada et al., 2015). Here *C. albicans* treated with the combination of AMB and FTY720 showed higher levels of oxidative enzymes than control group or single drug group, which corroborates the hypothesis that an adaptive response occurs when *Candida* is exposed to oxidative stress. Our results suggest that the combination of FTY720 and AMB exhibits an oxidant effect on *Candida*, resulting in the hypersensitivity of *Candida* to phagocytic cell attack (Arce Miranda et al., 2019).

*C. albicans* attaches and adheres to the epithelial cells through adhesins and invades epithelial cells by induced endocytosis and active penetration (Naglik et al., 2011; Wachtler et al., 2012). Induced endocytosis was normally initiated in the cell entry stage for further invasion (Naglik et al., 2011). This host-driven mechanism, which requires the host cell to actively participate in, is widespread in many microbe-epithelial interactions in microbial pathogens including bacteria and fungi (Goosney et al., 1999; Moyes et al., 2015). Since low concentration of FTY720 shows no cytotoxicity to mammalian cells, we performed the endocytosis assay and found that the endocytosis of *C. albicans* by FaDu oral epithelial cells was reduced when combined with AMB (*P* <0.001), which may be due to the inhibitory effect of the drug combination on the viability, activity and invasion of *C. albicans*. Recruitment of phagocytes to the infection site plays an important role in the body’s innate immune defense against *C. albicans* (Sheth et al., 2011). *C. albicans* cocultured with phagocytic cells under the combination of FTY720 and AMB (**Figure S7**) turned out to have less viability and to be more susceptible to phagocytic cell killing indicating better antifungal efficacy of the combination of FTY720 and AMB.

A major mechanism of killing engulfed fungi by innate immune defender phagocytes is the NADPH-oxidase complex (NOX2)-mediated process, a respiratory burst (also called an oxidative burst) (Dantas Ada et al., 2015; Salvatori et al., 2018). NOX2 assembles on the membrane of maturing neutrophil phagosomes and can produce ROS and reactive nitrogen species (RNS) to defend against phagocytosed microbes (Dantas Ada et al., 2015; Salvatori et al., 2018). Interestingly, *C. albicans* can immediately induce the expression of antioxidant proteins including CAT, SOD and glutathione peroxidase to cope with the oxidative stress induced by the pathogen-host interaction, which plays an important role in its tolerance to high oxidative stress, resistance to phagocyte killing, immune evasion from the host immune response and survival in the host (Frohner et al., 2009). However, the ROS produced by NOX2 can also be secreted outside phagosomes to cause an oxidative stress of *C. albicans* prior to phagocytosis (Frohner et al., 2009; Miramon et al., 2012), which will recruit more phagocytes to the infection site (Brothers et al., 2013), limit filamentous growth intracellularly (Brothers et al., 2013) and form new toxic substances with other chemicals (Brown et al., 2009), eventually forming a fungicidal environment for *C. albicans* (Dantas Ada et al., 2015). Additionally, *C. albicans* is susceptible to the combination of ROS and cationic fluxes, which also accounts for the potent antifungal efficacy of phagocyte (Kaloriti et al., 2014). Cations possesses the potential to inhibit the detoxification of H_2_O_2_ resulting in intracellular hyperaccumulation of ROS (Kaloriti et al., 2014). It was reported that proteases, including elastase and cathepsin G, activated by K^+^ flux contributed to killing activity of phagocytes (Reeves et al., 2002). In our study, the combinatorial FTY720 and AMB provided assistance to the phagocyte in the battle against invasive fungi.

This study indicated for the first time that the FTY720 was able to synergize with AMB against *Candida* species, which offers the possibility to decrease the toxicity of the high dose of FTY720 or AMB and to improve its therapeutic efficacy against fungal infection in IBD patients. We also further determined the oxidative stress-induced effect of FTY720 and AMB in *C. albicans*. However, these *in vitro* results are not enough to support the application of the combinatorial drugs in clinical, which needs *in vivo* studies and even structural modification of FTY720. Further studies are needed to investigate the existence of a synergism of FTY720 and AMB in the murine mouse model of candidiasis, as well as the exact molecular mechanism underlying the synergistic antifungal effect. In conclusion, our investigation provided a new therapeutic strategy against fungal infection.

## Data Availability Statement

The original contributions presented in the study are included in the article/**Supplementary Material**. Further inquiries can be directed to the corresponding authors.

## Author Contributions

N-NL, HW, and J-QL conceived and designed the study. N-NL, L-QW, J-CT, YW, and Y-KM wrote the manuscript and discussed the experiments and results. L-QW, J-CT, YW, Y-KM, J-YX, LT, and K-YS conducted all experiments and performed the statistical analysis of the data. Y-CC and Y-BP kindly provided clinical isolated strains from Ruijin Hospital. All authors contributed to the article and approved the submitted version.

## Funding

This study was supported by grants from the National Key R&D Program of China (2020YFA0907200, 2018YFC2000700), National Natural Science Foundation of China (81572053, 81630086, 81971993, 31900129), the Key Research Program (ZDRW-ZS-2017-1) of the Chinese Academy of Sciences, the Major Science and Technology Innovation Program of Shanghai Municipal Education Commission (2019-01-07-00-01-E00059), the Program for Young Eastern Scholar at Shanghai Institutions of Higher Learning program (QD2018016), Shanghai Pujiang Program (18PJ1406600), Medicine and Engineering Interdisciplinary Research Fund of Shanghai Jiao Tong University (YG2020YQ06, YG2020YQ19, YG2019QNB39), Innovative research team of high-level local universities in Shanghai (SSMU-ZLCX20180302).

## Conflict of Interest

The authors declare that the research was conducted in the absence of any commercial or financial relationships that could be construed as a potential conflict of interest.

## References

[B1] Achkar J. M. Fries B. C. (2010). Candida Infections of the Genitourinary Tract. Clin. Microbiol. Rev. 23, 253–273. 10.1128/CMR.00076-09 20375352 PMC2863365

[B2] Arce Miranda J. E. Baronetti J. L. Sotomayor C. E. Paraje M. G. (2019). Oxidative and Nitrosative Stress Responses During Macrophage-Candida Albicans Biofilm Interaction. Med. Mycol. 57, 101–113. 10.1093/mmy/myx143 29294039

[B3] Belenky P. Camacho D. Collins J. J. (2013). Fungicidal Drugs Induce a Common Oxidative-Damage Cellular Death Pathway. Cell Rep. 3, 350–358. 10.1016/j.celrep.2012.12.021 23416050 PMC3656588

[B4] Brothers K. M. Gratacap R. L. Barker S. E. Newman Z. R. Norum A. Wheeler R. T. (2013). NADPH Oxidase-Driven Phagocyte Recruitment Controls Candida Albicans Filamentous Growth and Prevents Mortality. PloS Pathog. 9, e1003634. 10.1371/journal.ppat.1003634 24098114 PMC3789746

[B5] Brown A. J. Haynes K. Quinn J. (2009). Nitrosative and Oxidative Stress Responses in Fungal Pathogenicity. Curr. Opin. Microbiol. 12, 384–391. 10.1016/j.mib.2009.06.007 19616469 PMC2728829

[B6] Carter C. D. Kitchen L. E. Au W. C. Babic C. M. Basrai M. A. (2005). Loss of SOD1 and LYS7 Sensitizes Saccharomyces Cerevisiae to Hydroxyurea and DNA Damage Agents and Downregulates MEC1 Pathway Effectors. Mol. Cell Biol. 25, 10273–10285. 10.1128/MCB.25.23.10273-10285.2005 16287844 PMC1291217

[B7] Chowdhury T. Kohler J. R. (2015). Ribosomal Protein S6 Phosphorylation is Controlled by TOR and Modulated by PKA in Candida Albicans. Mol. Microbiol. 98, 384–402. 10.1111/mmi.13130 26173379 PMC4631378

[B8] Clinical Laboratory Standards Institute (2008). Reference Method for Broth Dilution Antifungal Susceptibility Testing of Yeast, Approved Standard – 3rd Edn, CLSI document M27-A3. Wayne: CLSI.

[B9] Danese S. Furfaro F. Vetrano S. (2018). Targeting S1P in Inflammatory Bowel Disease: New Avenues for Modulating Intestinal Leukocyte Migration. J. Crohn’s Colitis 12, S678–S686. 10.1093/ecco-jcc/jjx107 28961752

[B10] Dantas Ada S. Day A. Ikeh M. Kos I. Achan B. Quinn J. (2015). Oxidative Stress Responses in the Human Fungal Pathogen, Candida Albicans. Biomolecules 5, 142–165. 10.3390/biom5010142 25723552 PMC4384116

[B11] Feng Z. Zhou H. Ma S. Guan X. Chen L. Huang J.. (2018). FTY720 Attenuates Intestinal Injury and Suppresses Inflammation in Experimental Necrotizing Enterocolitis Via Modulating CXCL5/CXCR2 Axis. Biochem. Biophys. Res. Commun. 505, 1032–1037. 10.1016/j.bbrc.2018.10.013 30314693

[B12] Ferreira G. F. Baltazar Lde M. Santos J. R. Monteiro A. S. Fraga L. A. Resende-Stoianoff M. A.. (2013). The Role of Oxidative and Nitrosative Bursts Caused by Azoles and Amphotericin B Against the Fungal Pathogen Cryptococcus Gattii. J. Antimicrob. Chemother. 68, 1801–1811. 10.1093/jac/dkt114 23612570

[B13] François I. E. J. A. Cammue B. P. A. Borgers M. Ausma J. Gerrit D. D. (2006). Azoles: Mode of Antifungal Action and Resistance Development. Effect of Miconazole on Endogenous Reactive Oxygen Species Production in Candida Albicans. Anti-Infect. Agents Med. Chem. 5, 3–13. 10.2174/187152106774755554

[B14] Frohner I. E. Bourgeois C. Yatsyk K. Majer O. Kuchler K. (2009). Candida Albicans Cell Surface Superoxide Dismutases Degrade Host-Derived Reactive Oxygen Species to Escape Innate Immune Surveillance. Mol. Microbiol. 71, 240–252. 10.1111/j.1365-2958.2008.06528.x 19019164 PMC2713856

[B15] Gerard R. Sendid B. Colombel J.-F. Poulain D. Jouault T. (2015). An Immunological Link Between Candida Albicans Colonization and Crohn’s Disease. Crit. Rev. Microbiol. 41, 135–139. 10.3109/1040841X.2013.810587 23855357

[B16] Ghannoum M. A. Rice L. B. (1999). Antifungal Agents: Mode of Action, Mechanisms of Resistance, and Correlation of These Mechanisms With Bacterial Resistance. Clin. Microbiol. Rev. 12, 501–517. 10.1128/CMR.12.4.501 10515900 PMC88922

[B17] Goosney D. L. Knoechel D. G. Finlay B. B. (1999). Enteropathogenic E. Coli, Salmonella, and Shigella: Masters of Host Cell Cytoskeletal Exploitation. Emerg. Infect. Dis. 5, 216–223. 10.3201/eid0502.990205 10221873 PMC2640686

[B18] Guirao-Abad J. P. Sánchez-Fresneda R. Alburquerque B. Hernández J. A. Argüelles J. C. (2017). ROS Formation is a Differential Contributory Factor to the Fungicidal Action of Amphotericin B and Micafungin in Candida Albicans. Int. J. Med. Microbiol. 307, 241–248. 10.1016/j.ijmm.2017.03.005 28412040

[B19] Gulati M. Nobile C. J. (2016). Candida Albicans Biofilms: Development, Regulation, and Molecular Mechanisms. Microbes Infect. 18, 310–321. 10.1016/j.micinf.2016.01.002 26806384 PMC4860025

[B20] Hager C. L. Ghannoum M. A. (2017). The Mycobiome: Role in Health and Disease, and as a Potential Probiotic Target in Gastrointestinal Disease. Digest Liver Dis. Off. J. Ital. Soc. Gastroenterol. Ital. Assoc. Study Liver 49, 1171–1176. 10.1016/j.dld.2017.08.025 28988727

[B21] Huwiler A. Zangemeister-Wittke U. (2018). The Sphingosine 1-Phosphate Receptor Modulator Fingolimod as a Therapeutic Agent: Recent Findings and New Perspectives. Pharmacol. Ther. 185, 34–49. 10.1016/j.pharmthera.2017.11.001 29127024

[B22] Jin Y. Yip H. K. Samaranayake Y. H. Yau J. Y. Samaranayake L. P. (2003). Biofilm-Forming Ability of Candida Albicans is Unlikely to Contribute to High Levels of Oral Yeast Carriage in Cases of Human Immunodeficiency Virus Infection. J. Clin. Microbiol. 41, 2961–2967. 10.1128/JCM.41.7.2961-2967.2003 12843027 PMC165379

[B23] Johnson M. D. Macdougall C. Ostrosky-Zeichner L. Perfect J. R. Rex J. H. (2004). Combination Antifungal Therapy. Antimicrob. Agents Chemother. 48, 693–715. 10.1128/AAC.48.3.693-715.2004 14982754 PMC353116

[B24] Kaloriti D. Jacobsen M. Yin Z. Patterson M. Tillmann A. Smith D. A.. (2014). Mechanisms Underlying the Exquisite Sensitivity of Candida Albicans to Combinatorial Cationic and Oxidative Stress That Enhances the Potent Fungicidal Activity of Phagocytes. mBio 5, e01334–e01314. 10.1128/mBio.01334-14 25028425 PMC4161263

[B25] Khan M. S. Ahmad I. (2012). Antibiofilm Activity of Certain Phytocompounds and Their Synergy With Fluconazole Against Candida Albicans Biofilms. J. Antimicrob. Chemother. 67, 618–621. 10.1093/jac/dkr512 22167241

[B26] Kim J. K. Park J. Ryu T. H. Nili M. (2013). Effect of N-Acetyl-L-Cysteine on Saccharomyces Cerevisiae Irradiated With Gamma-Rays. Chemosphere 92, 512–516. 10.1016/j.chemosphere.2013.02.035 23623538

[B27] Lewis R. E. Diekema D. J. Messer S. A. Pfaller M. A. Klepser M. E. (2002). Comparison of Etest, Chequerboard Dilution and Time-Kill Studies for the Detection of Synergy or Antagonism Between Antifungal Agents Tested Against Candida Species. J. Antimicrob. Chemother. 49, 345–351. 10.1093/jac/49.2.345 11815578

[B28] Li J. Chen D. Yu B. He J. Zheng P. Mao X.. (2018). Fungi in Gastrointestinal Tracts of Human and Mice: From Community to Functions. Microbial Ecol. 75, 821–829. 10.1007/s00248-017-1105-9 29110065

[B29] Li H. Z. Guo J. Gao J. Han L. P. Jiang C. M. Li H. X.. (2011). Role of Dopamine D2 Receptors in Ischemia/Reperfusion Induced Apoptosis of Cultured Neonatal Rat Cardiomyocytes. J. BioMed. Sci. 18, 18. 10.1186/1423-0127-18-18 21324201 PMC3050795

[B30] Li L. Zhang T. Xu J. Wu J. Wang Y. Qiu X.. (2019). The Synergism of the Small Molecule Enoblock and Fluconazole Against Fluconazole-Resistant Candida Albicans. Front. Microbiol. 10, 2071. 10.3389/fmicb.2019.02071 31555252 PMC6742966

[B31] Liu N. N. Flanagan P. R. Zeng J. Jani N. M. Cardenas M. E. Moran G. P.. (2017). Phosphate is the Third Nutrient Monitored by TOR in Candida Albicans and Provides a Target for Fungal-Specific Indirect TOR Inhibition. Proc. Natl. Acad. Sci. U.S.A. 114, 6346–6351. 10.1073/pnas.1617799114 28566496 PMC5474788

[B32] Liu N. N. Uppuluri P. Broggi A. Besold A. Ryman K. Kambara H.. (2018). Intersection of Phosphate Transport, Oxidative Stress and TOR Signalling in Candida Albicans Virulence. PloS Pathog. 14, e1007076. 10.1371/journal.ppat.1007076 30059535 PMC6085062

[B33] Martinez-Lopez R. Park H. Myers C. L. Gil C. Filler S. G. (2006). Candida Albicans Ecm33p is Important for Normal Cell Wall Architecture and Interactions With Host Cells. Eukaryot Cell 5, 140–147. 10.1128/EC.5.1.140-147.2006 16400176 PMC1360258

[B34] Mei Y. Jiang T. Zou Y. Wang Y. Zhou J. Li J.. (2020). FDA Approved Drug Library Screening Identifies Robenidine as a Repositionable Antifungal. Front. Microbiol. 11, 996. 10.3389/fmicb.2020.00996 32582050 PMC7283467

[B35] Mesa-Arango A. C. Trevijano-Contador N. Roman E. Sanchez-Fresneda R. Casas C. Herrero E.. (2014). The Production of Reactive Oxygen Species is a Universal Action Mechanism of Amphotericin B Against Pathogenic Yeasts and Contributes to the Fungicidal Effect of This Drug. Antimicrob. Agents Chemother. 58, 6627–6638. 10.1128/AAC.03570-14 25155595 PMC4249417

[B36] Miramon P. Dunker C. Windecker H. Bohovych I. M. Brown A. J. Kurzai O.. (2012). Cellular Responses of Candida Albicans to Phagocytosis and the Extracellular Activities of Neutrophils are Critical to Counteract Carbohydrate Starvation, Oxidative and Nitrosative Stress. PloS One 7, e52850. 10.1371/journal.pone.0052850 23285201 PMC3528649

[B37] Moyes D. L. Richardson J. P. Naglik J. R. (2015). Candida Albicans-Epithelial Interactions and Pathogenicity Mechanisms: Scratching the Surface. Virulence 6, 338–346. 10.1080/21505594.2015.1012981 25714110 PMC4601190

[B38] Mukherjee P. K. Sheehan D. J. Hitchcock C. A. Ghannoum M. A. (2005). Combination Treatment of Invasive Fungal Infections. Clin. Microbiol. Rev. 18, 163–194. 10.1128/CMR.18.1.163-194.2005 15653825 PMC544182

[B39] Naglik J. R. Moyes D. L. Wächtler B. Hube B. (2011). Candida Albicans Interactions With Epithelial Cells and Mucosal Immunity. Microbes Infect. 13, 963–976. 10.1016/j.micinf.2011.06.009 21801848 PMC3185145

[B40] Nakagawa Y. (2008). Catalase Gene Disruptant of the Human Pathogenic Yeast Candida Albicans is Defective in Hyphal Growth, and a Catalase-Specific Inhibitor Can Suppress Hyphal Growth of Wild-Type Cells. Microbiol. Immunol. 52, 16–24. 10.1111/j.1348-0421.2008.00006.x 18352908

[B41] Nobile C. J. Johnson A. D. (2015). Candida Albicans Biofilms and Human Disease. Annu. Rev. Microbiol. 69, 71–92. 10.1146/annurev-micro-091014-104330 26488273 PMC4930275

[B42] Nooney L. Matthews R. C. Burnie J. P. (2005). Evaluation of Mycograb, Amphotericin B, Caspofungin, and Fluconazole in Combination Against Cryptococcus Neoformans by Checkerboard and Time-Kill Methodologies. Diagn. Microbiol. Infect. Dis. 51, 19–29. 10.1016/j.diagmicrobio.2004.08.013 15629225

[B43] Odds F. C. (2003). Synergy, Antagonism, and What the Chequerboard Puts Between Them. J. Antimicrob. Chemother. 52, 1. 10.1093/jac/dkg301 12805255

[B44] Panés J. Salas A. (2018). Past, Present and Future of Therapeutic Interventions Targeting Leukocyte Trafficking in Inflammatory Bowel Disease. J. Crohn’s Colitis 12, S633–S640. 10.1093/ecco-jcc/jjy011 30137311

[B45] Pinschewer D. D. Ochsenbein A. F. Odermatt B. Brinkmann V. Hengartner H. Zinkernagel R. M. (2000). FTY720 Immunosuppression Impairs Effector T Cell Peripheral Homing Without Affecting Induction, Expansion, and Memory. J. Immunol. 164, 5761–5770. 10.4049/jimmunol.164.11.5761 10820254

[B46] Prieto D. Correia I. Pla J. Román E. (2016). Adaptation of Candida Albicans to Commensalism in the Gut. Future Microbiol. 11, 567–583. 10.2217/fmb.16.1 27070839

[B47] Reeves E. P. Lu H. Jacobs H. L. Messina C. G. Bolsover S. Gabella G.. (2002). Killing Activity of Neutrophils is Mediated Through Activation of Proteases by K+ Flux. Nature 416, 291–297. 10.1038/416291a 11907569

[B48] Salvatori O. Pathirana R. U. Kay J. G. Edgerton M. (2018). Candida Albicans Ras1 Inactivation Increases Resistance to Phagosomal Killing by Human Neutrophils. Infect. Immun. 86, e00685-18. 10.1128/IAI.00685-18 30249746 PMC6246910

[B49] Sarkar S. Uppuluri P. Pierce C. G. Lopez-Ribot J. L. (2014). In Vitro Study of Sequential Fluconazole and Caspofungin Treatment Against Candida Albicans Biofilms. Antimicrob. Agents Chemother. 58, 1183–1186. 10.1128/AAC.01745-13 24217700 PMC3910858

[B50] Scherz-Shouval R. Elazar Z. (2007). ROS, Mitochondria and the Regulation of Autophagy. Trends Cell Biol. 17, 422–427. 10.1016/j.tcb.2007.07.009 17804237

[B51] Sheth C. C. Hall R. Lewis L. Brown A. J. Odds F. C. Erwig L. P.. (2011). Glycosylation Status of the C. Albicans Cell Wall Affects the Efficiency of Neutrophil Phagocytosis and Killing But Not Cytokine Signaling. Med. Mycol. 49, 513–524. 10.3109/13693786.2010.551425 21254968 PMC3119872

[B52] Silva L. N. Oliveira S. S. C. Magalhaes L. B. Andrade Neto V. V. Torres-Santos E. C. Carvalho M. D. C.. (2020). Unmasking the Amphotericin B Resistance Mechanisms in Candida Haemulonii Species Complex. ACS Infect. Dis. 6, 1273–1282. 10.1021/acsinfecdis.0c00117 32239912

[B53] Stamatiades G. A. Ioannou P. Petrikkos G. Tsioutis C. (2018). Fungal Infections in Patients With Inflammatory Bowel Disease: A Systematic Review. Mycoses 61, 366–376. 10.1111/myc.12753 29453860 PMC5980782

[B54] Sukocheva O. A. Furuya H. Ng M. L. Friedemann M. Menschikowski M. Tarasov V. V.. (2020). Sphingosine Kinase and Sphingosine-1-Phosphate Receptor Signaling Pathway in Inflammatory Gastrointestinal Disease and Cancers: A Novel Therapeutic Target. Pharmacol. Ther. 207, 107464. 10.1016/j.pharmthera.2019.107464 31863815

[B55] Vonk A. G. Wieland C. W. Netea M. G. Kullberg B. J. (2002). Phagocytosis and Intracellular Killing of Candida Albicans Blastoconidia by Neutrophils and Macrophages: A Comparison of Different Microbiological Test Systems. J. Microbiol. Methods 49, 55–62. 10.1016/S0167-7012(01)00348-7 11777582

[B56] Wachtler B. Citiulo F. Jablonowski N. Forster S. Dalle F. Schaller M.. (2012). Candida Albicans-Epithelial Interactions: Dissecting the Roles of Active Penetration, Induced Endocytosis and Host Factors on the Infection Process. PloS One 7, e36952. 10.1371/journal.pone.0036952 22606314 PMC3351431

[B57] Yu Q. Zhang B. Li J. Zhang B. Wang H. Li M. (2016). Endoplasmic Reticulum-Derived Reactive Oxygen Species (ROS) is Involved in Toxicity of Cell Wall Stress to Candida Albicans. Free Radic. Biol. Med. 99, 572–583. 10.1016/j.freeradbiomed.2016.09.014 27650297

