# Supplementary material for: Fingolimod Potentiates the Antifungal Activity of Amphotericin B
Source: Front Cell Infect Microbiol. 2021 Apr 23;11:627917. doi: 10.3389/fcimb.2021.627917 (PMC8102868; doi:10.3389/fcimb.2021.627917)
Supplement: Supplementary file 1 [file DataSheet_1.docx]

Supplementary Material

# Supplementary Figures and Tables

## Supplementary Figures


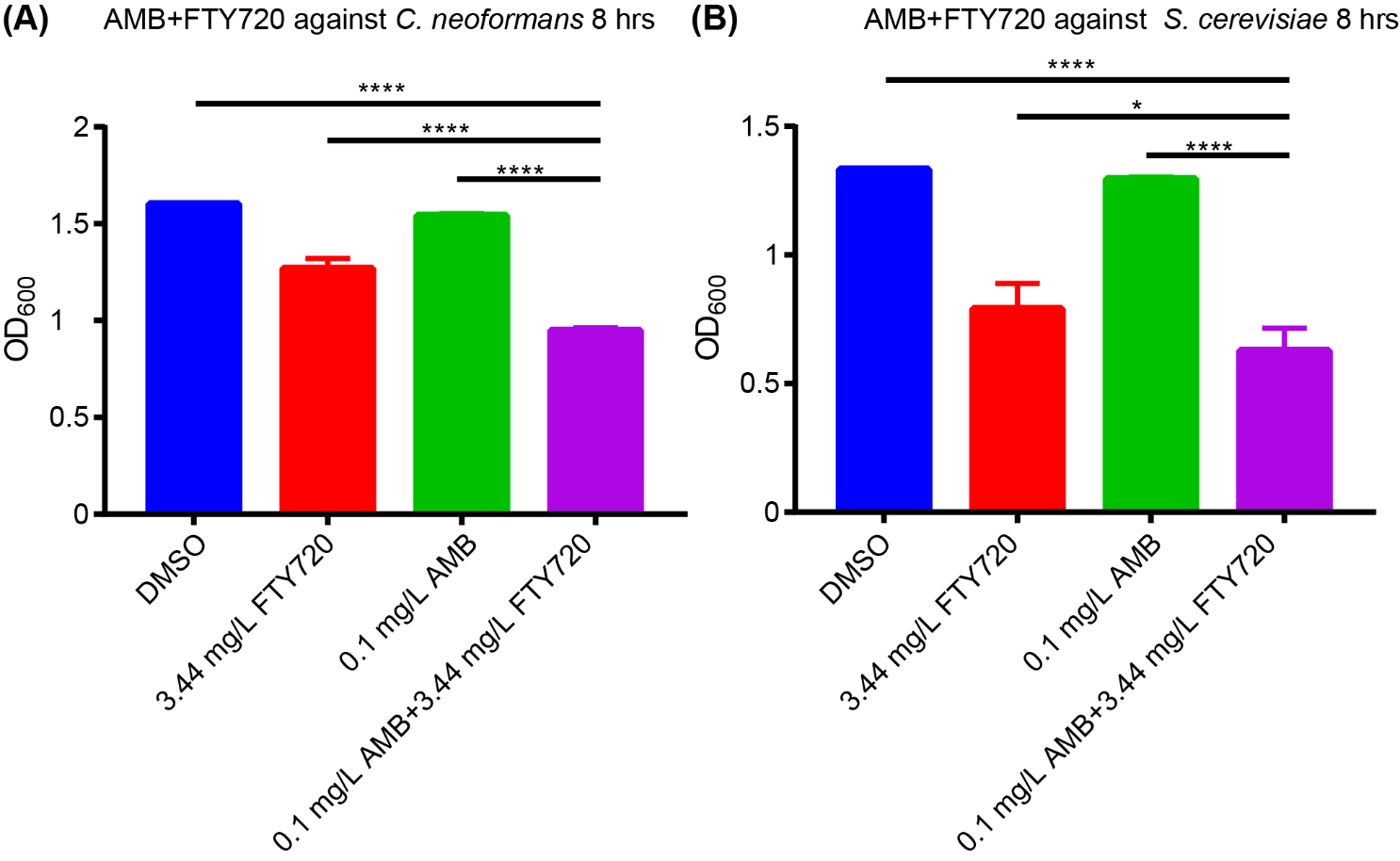


**Supplementary Figure 1.** Inhibitory effect on growth of *C. neoformans* (A) and *S. cerevisiae* (B) by 3.44 mg/L FTY720 and 0.1 mg/L AMB at 8 h. *P* values were calculated using one-way analysis of variance (ANOVA) with Dunnett’s corrected post-hoc comparisons. *, *P* < 0.05 vs AMB+FTY720; ****, *P* < 0.0001 vs AMB+FTY720.

**
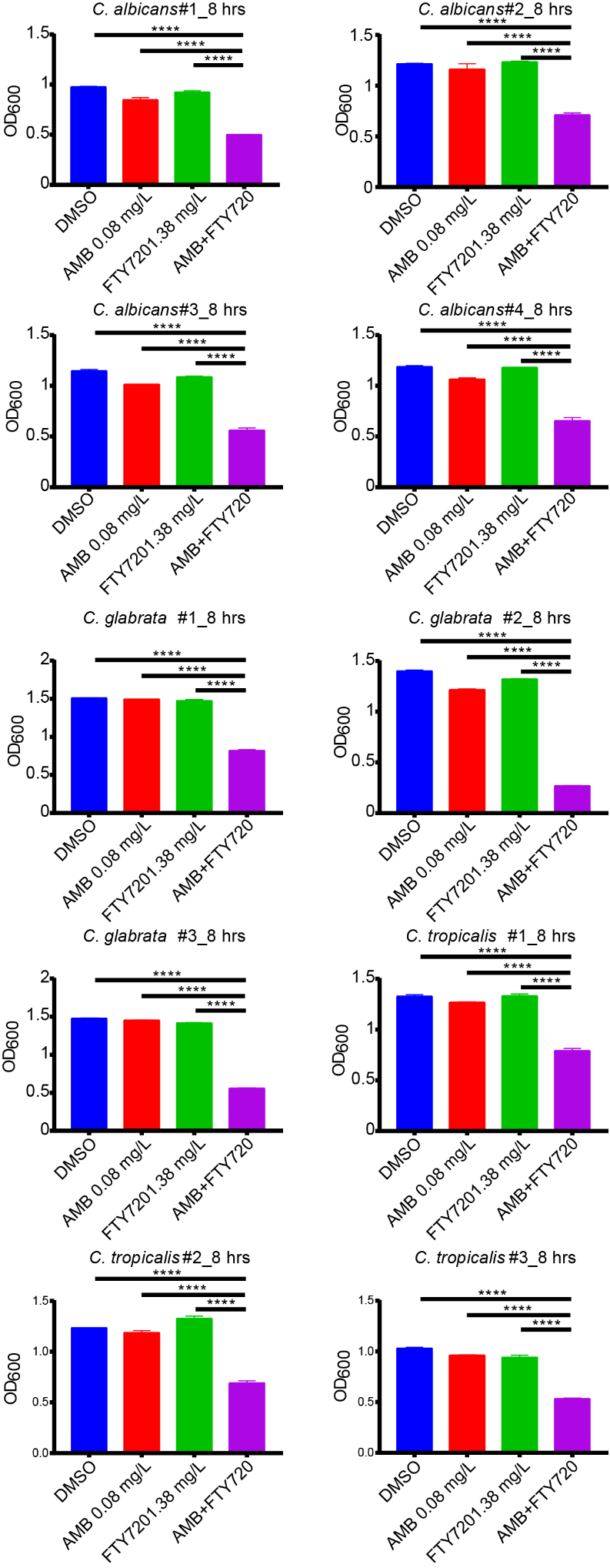
**

**Supplementary Figure 2.** Inhibitory effect on growth of different *Candida* clinical isolates by 1.38 mg/L FTY720 and 0.08 mg/L AMB at 8 h. *P* values were calculated using one-way analysis of variance (ANOVA) with Dunnett’s corrected post-hoc comparisons. ****, *P* < 0.0001 vs AMB+FTY720.


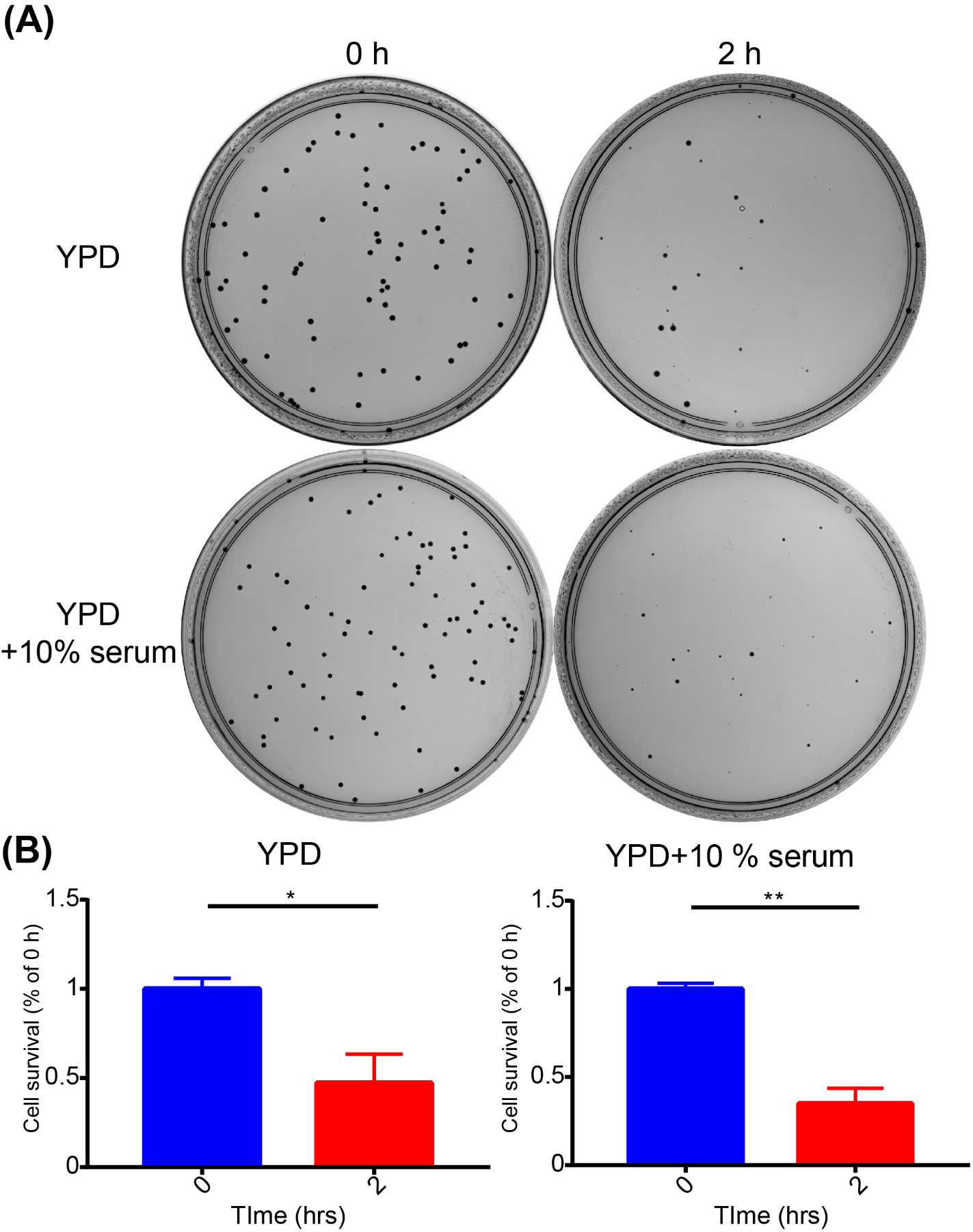


**Supplementary Figure 3.** Survival rate of *C. albicans* after treatment with FTY720. (A) Representative images of *C. albicans* CFU assay treated with 11 mg/L FTY720 at 0 h and 2 h. (B) Survival rate of *C. albicans* treated with 11 mg/L FTY720 in YPD or YPD + 10 % serum medium at 0 h and 2 h measured by CFU assay. *P* values were calculated using two-tailed unpaired Student’s *t*-test. *, *P* < 0.05; **, *P* < 0.01.


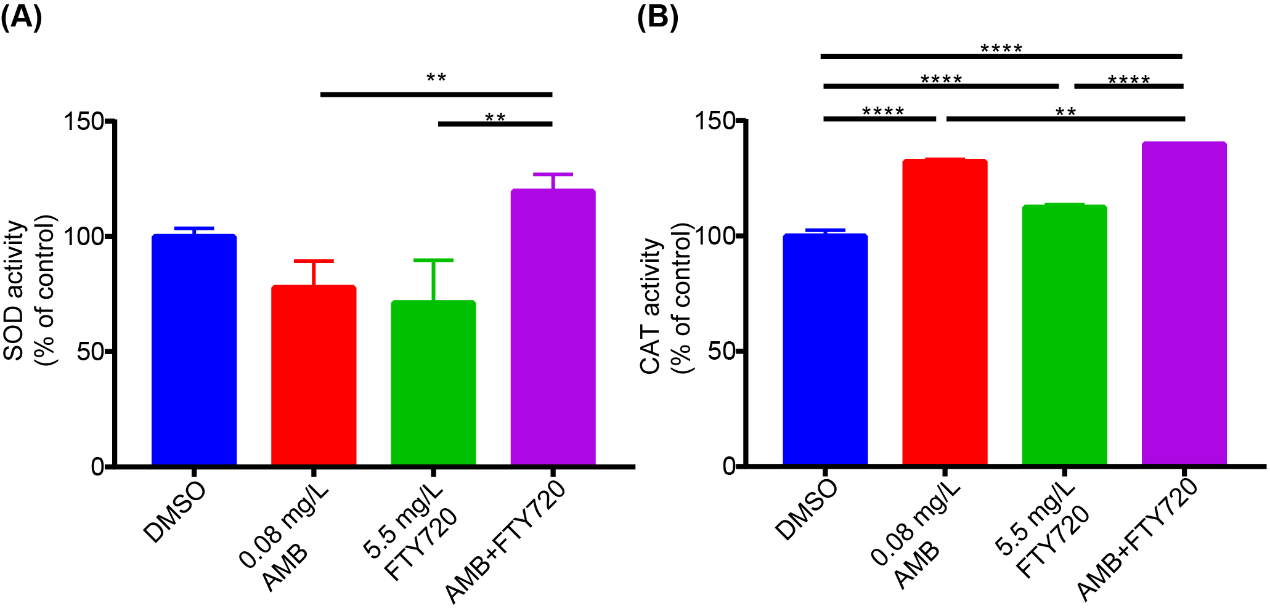


**Supplementary Figure 4.** The SOD activity (A) and CAT activity (B) in *C. albicans* cells after treatment with combination of 0.08 mg/L AMB and 5.5 mg/L FTY720. Results are shown as mean ± SD. *p* values were calculated using one-way analysis of variance (ANOVA) with Tukey’s corrected post-hoc comparisons. **, *P* < 0.01; ****, *P* < 0.0001.


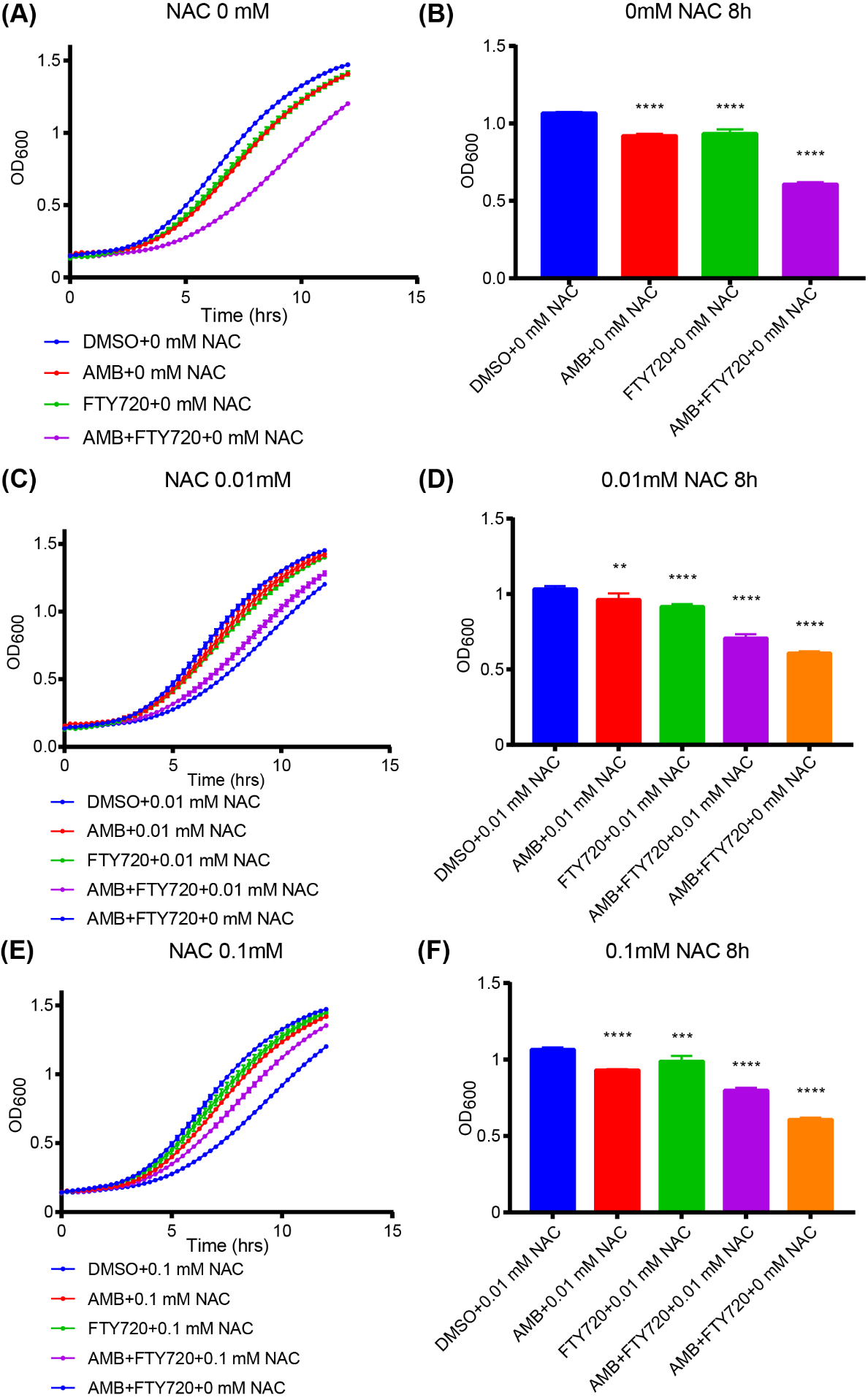


**Supplementary Figure 5.** The antifungal effects of AMB and FTY720 against *C. albicans* was enhanced by treatment with different concentrations of NAC. (A), (C) and (E) The cell concentration were adjusted to OD_600_=0.1 in YPD medium with single or combinatorial treatment with 0.08 mg/L AMB and 2.75 mg/L FTY720 with/without different concentrations of NAC. The OD_600_ was obtained every 15 min in a BioTek plate reader for 12 hours. Results are the mean ± SD of three experiments, and each was performed in triplicates. (B), (D) and (F) The OD_600_ were measured 8 hours post incubation. *P* values were calculated using one-way analysis of variance (ANOVA) with Dunnett’s corrected post-hoc comparisons. **, *P* < 0.01, ***, *P* < 0.001, ****, *P* < 0.0001 vs control.


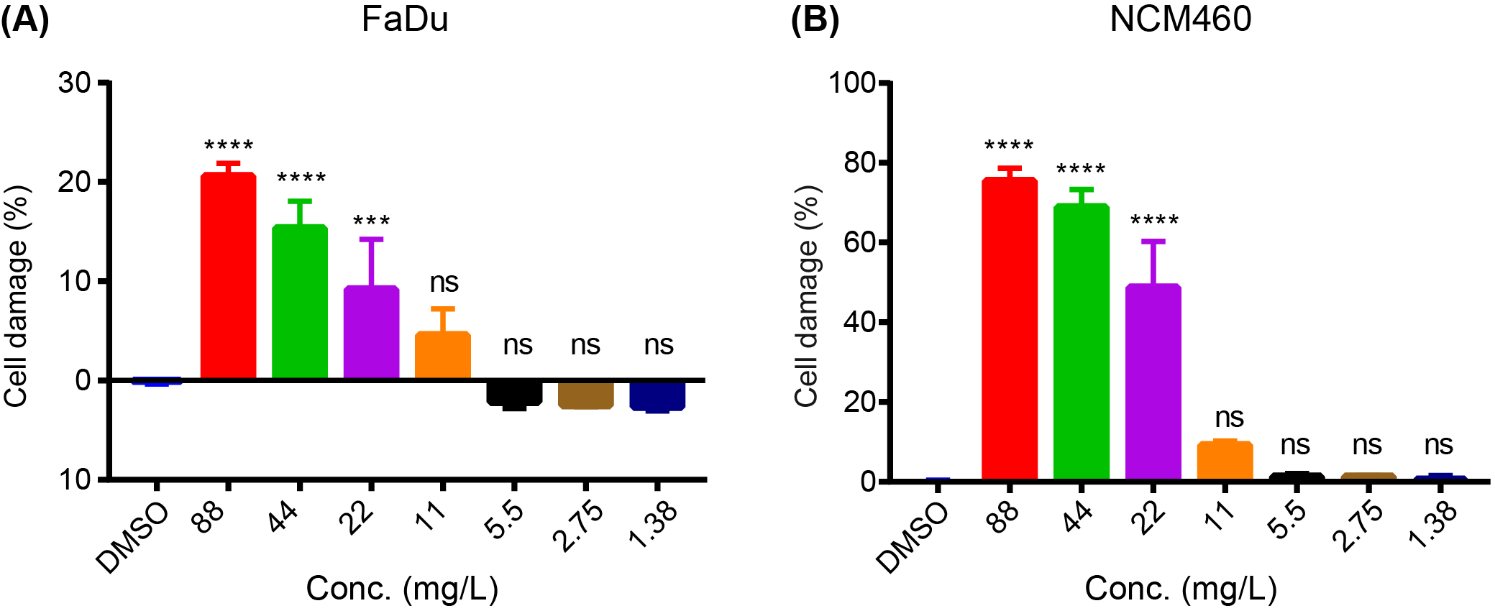


**Supplementary Figure 6.** The cytotoxicity of FTY720 on FaDu (A) and NCM460 cells. A total of 2 × 10^4^ FaDu cells (or 1 × 10^4^ NCM460 cells) were incubated with different concentration of FTY720 for 1 h. Results are shown as mean ± SD. *P* values were calculated using one-way analysis of variance (ANOVA) with Dunnett’s corrected post-hoc comparisons. ***, *P* < 0.001, ****, *P* < 0.0001 vs control.


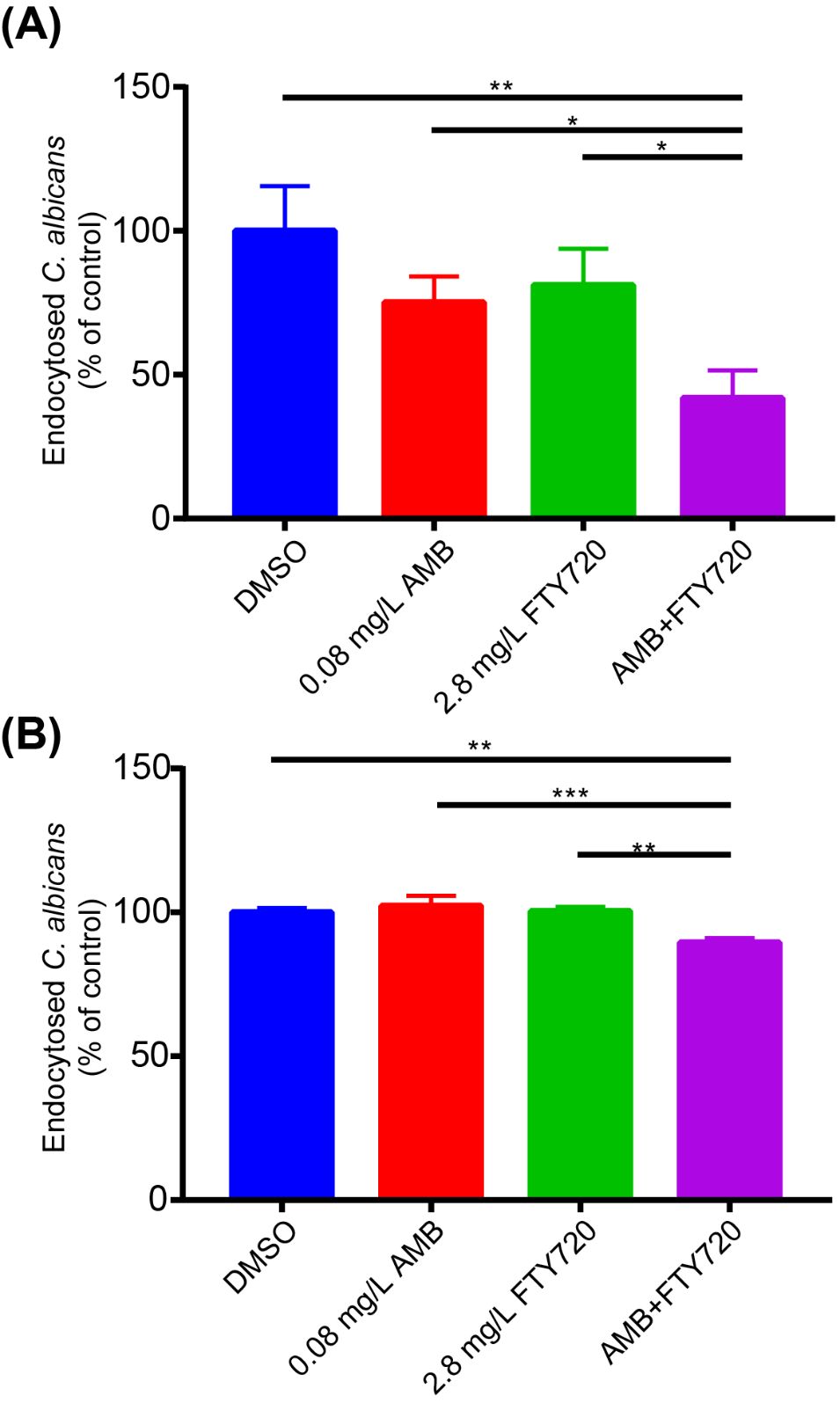


**Supplementary Figure 7.** Effects on the interaction between *C. albicans* and host cells after treatment with FTY720 and AMB. (A) Effects of inhibition of FTY720 and AMB on epithelial cell endocytosis of *C. albicans*. (B) Percent survival of *C. albicans* cells after incubation with macrophage treated with 2.8 mg/L FTY720 and 0.08 mg/L AMB. Results are shown as mean ± SD. *P* values were calculated using one-way analysis of variance (ANOVA) with Tukey’s corrected post-hoc comparisons. *, *P* < 0.05; **, *P* < 0.01; ***, *P* < 0.001.

## Supplementary Tables

**Supplementary Table 1**. Strains used in this study

| Strain name | Description | Reference |
| --- | --- | --- |
| SC5314 | WT strain of *Candida albicans*  Gift from Dr. Julia R. Koehler | (Romo et al., 2019) |
| BY4742 | WT strain of *Saccharomyces cerevisiae*  Gift from Dr. Jin-Qiu Zhou | (Wang et al., 2011) |
| H99 | WT strain of *Cryptococcus neoformans*  Gift from Dr. Changbin Chen | (Li et al., 2019) |

| Strains | Number | Gender | Age | Sample type |
| --- | --- | --- | --- | --- |
| *C. albicans* | 1 | male | 85 | sputum |
| *C. albicans* | 2 | male | 65 | drainage |
| *C. albicans* | 3 | female | 50 | wound |
| *C. albicans* | 4 | male | 79 | drainage |
| *C. glabrata* | 1 | female | 22 | vaginal secretions |
| *C. glabrata* | 2 | female | 84 | sputum |
| *C. glabrata* | 3 | female | 29 | vaginal secretions |
| *C. tropicalis* | 1 | female | 47 | sputum |
| *C. tropicalis* | 2 | female | 47 | sputum |
| *C. tropicalis* | 3 | male | 93 | sputum |

**Supplementary Table 2.** Patients’ information of clinical strains used in this study

Li, Y., Li, H., Sui, M., Li, M., Wang, J., Meng, Y., et al. (2019). Fungal acetylome comparative analysis identifies an essential role of acetylation in human fungal pathogen virulence. *Commun Biol* 2**,** 154. doi: 10.1038/s42003-019-0419-1.

Romo, J.A., Zhang, H., Cai, H., Kadosh, D., Koehler, J.R., Saville, S.P., et al. (2019). Global Transcriptomic Analysis of the Candida albicans Response to Treatment with a Novel Inhibitor of Filamentation. *mSphere* 4(5). doi: 10.1128/mSphere.00620-19.

Wang, S.S., Zhou, B.O., and Zhou, J.Q. (2011). Histone H3 lysine 4 hypermethylation prevents aberrant nucleosome remodeling at the PHO5 promoter. *Mol Cell Biol* 31(15)**,** 3171-3181. doi: 10.1128/mcb.05017-11.

**References**
